# Interplay Between Gluten, HLA, Innate and Adaptive Immunity Orchestrates the Development of Coeliac Disease

**DOI:** 10.3389/fimmu.2021.674313

**Published:** 2021-06-02

**Authors:** Jordan Voisine, Valérie Abadie

**Affiliations:** ^1^ Department of Medicine, The University of Chicago, Chicago, IL, United States; ^2^ Committee on Immunology, The University of Chicago, Chicago, IL, United States; ^3^ Section of Gastroenterology, Nutrition and Hepatology, The University of Chicago, Chicago, IL, United States

**Keywords:** coeliac disease, villous atrophy, gluten, transglutaminase 2, HLA-DQ2/8, T lymphocytes

## Abstract

Several environmental, genetic, and immune factors create a “perfect storm” for the development of coeliac disease: the antigen gluten, the strong association of coeliac disease with HLA, the deamidation of gluten peptides by the enzyme transglutaminase 2 (TG2) generating peptides that bind strongly to the predisposing HLA-DQ2 or HLA-DQ8 molecules, and the ensuing unrestrained T cell response. T cell immunity is at the center of the disease contributing to the inflammatory process through the loss of tolerance to gluten and the differentiation of HLA-DQ2 or HLA-DQ8-restricted anti-gluten inflammatory CD4^+^ T cells secreting pro-inflammatory cytokines and to the killing of intestinal epithelial cells by cytotoxic intraepithelial CD8^+^ lymphocytes. However, recent studies emphasize that the individual contribution of each of these cell subsets is not sufficient and that interactions between these different populations of T cells and the simultaneous activation of innate and adaptive immune pathways in distinct gut compartments are required to promote disease immunopathology. In this review, we will discuss how tissue destruction in the context of coeliac disease results from the complex interactions between gluten, HLA molecules, TG2, and multiple innate and adaptive immune components.

## Introduction

Coeliac disease (CeD) is a multifactorial intestinal immune-mediated disorder with autoimmune features that leads to inflammatory and destructive lesions in the proximal small intestine. CeD, similar to other organ-specific autoimmune disorders, is marked by its complexity both at the epidemiological and immunological levels, which translates into a spectrum of clinical manifestations (1). CeD is characterized by an infiltration of intraepithelial lymphocytes in the proximal part of the small intestine, crypt hyperplasia and the development of villous atrophy in the latest stages of the disease. In addition, CeD patients produce highly disease-specific antibodies against deamidated gluten peptides and the enzyme tissue transglutaminase 2 (TG2) (2–4). CeD is triggered by gluten consumption in genetically susceptible individuals carrying certain major histocompatibility complex (MHC) class II human leukocyte antigen (HLA) variants (5, 6). 90-95% of CeD patients carry the HLA-DQ2.5 variant (DQA1*05:01, DQB1*02:01) that confers the highest risk of developing CeD while the remaining patients carry HLA-DQ2.2 (DQA1*02:01, DQB1*02:02) or HLA-DQ8 (DQA1*03, DQB1*03:02) (5, 7). However, while up to 40% of the general population in Western countries express one of the predisposing HLA molecules, the global prevalence of CeD is just 1% (8). This finding suggests that these HLA variants contribute to, but are not sufficient for, the development of the disease and that additional genetic and environmental factors are needed to mount a pathogenic immune response against gluten (9). In fact, the HLA locus which is the main inherited genetic susceptibility factor for CeD, only accounts for ~ 40% of the genetic variance of the disease. Hence, non-MHC susceptibility loci explaining ~ 15% of the disease risk (10–13), as well as additional environmental factors other than gluten, are thought to contribute to disease development. Among them are early life gastrointestinal infections, which have been associated with an increased risk of developing CeD in several cohorts of genetically susceptible children (14–16). Of particular interest are enteric viruses such as reovirus, norovirus and rotavirus, which are the most common causes of diarrheal disease in early childhood. Recurrent infections in young individuals with a permissive genetic background could interfere with the maturation of the mucosal immune system and the composition of the microbiome (17), and thus favor the subsequent induction of an inflammatory T cell responses and the loss of oral tolerance to dietary gluten (18, 19). Although much less documented, intestinal infections caused by bacteria such as *Campylobacter jejuni* or parasites such as *Giardia lamblia*, could also contribute to the onset or maintenance of the disease (20, 21). In strong support of a role of the microbial environment in promoting the development of CeD is the identification of microbially derived mimics of gliadin epitopes that can activate HLA-DQ2.5-restricted gliadin-specific T cells isolated from CeD patients (22).

**Figure 1:**
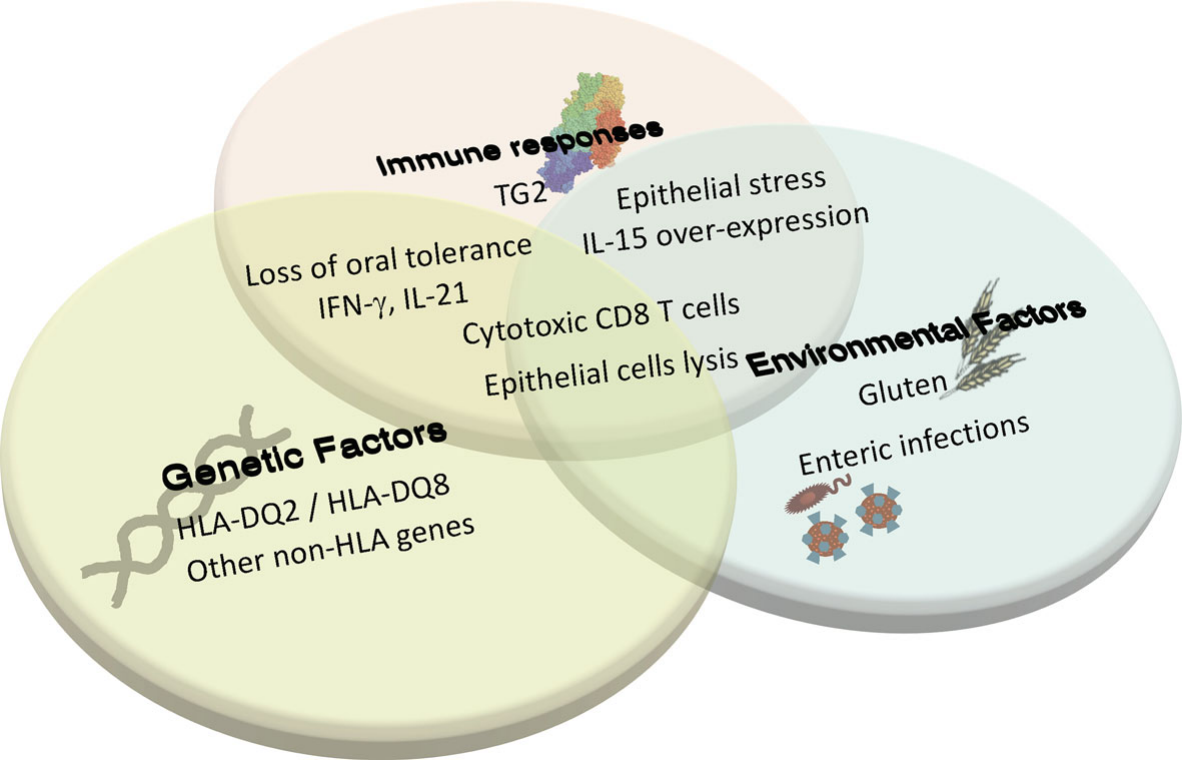
Coeliac disease is a multifactorial complex autoimmune disorder that requires the interplay between genetics, innate and adaptive immunity, and environmental triggers to cause tissue destruction. In individuals with HLA-DQ2/DQ8, induction of adaptive immune response against gluten and the loss of oral tolerance to gluten can occur when there is environmentally triggered epithelial stress and IL-15 overexpression. This adaptive response to gluten and the associated cytokine production promotes further tissue stress, leading to the licensing of cytotoxic CD8 T cells to lyse epithelial cells and cause villous atrophy. Additional environmental triggers and immune factors yet to be determined are also thought to contribute to disease development.

The complexity of CeD is also reflected by the contribution of multiple immune pathways for the induction of the disease and intestinal tissue remodeling and destruction (23–25). It has been known for decades that HLA-DQ2 and HLA-DQ8 present TG2-deamidated gluten peptides to CD4^+^ T cells in the intestinal lamia propria compartment, driving T_H_1 differentiation (26–31). These gluten-specific T_H_1 cells contribute to the inflammatory process through the production of the inflammatory cytokines Interferon (IFN)-γ (32) and Interleukin (IL)-21 (33). Yet, this gluten-specific adaptive immunity is not sufficient to promote the licensing of intraepithelial cytotoxic CD8^+^ T cells (IE-CTLs) that are responsible for the destruction of distressed intestinal epithelial cells. This lack of sufficiency can be seen in potential CeD patients, who carry HLA-DQ2 or HLA-DQ8 and display adaptive immune responses against gluten (proxied by anti-TG2 and anti-endomysium antibodies) but lack villous atrophy (6, 34). In particular, potential CeD patients do not display an accumulation of IE-CTLs with an active killer phenotype (upregulated granzyme B expression, upregulated activating NK receptor expression, downregulated inhibitory NK receptor expression) and also lack upregulation of the pro-inflammatory cytokine IL-15 and the non-classical MHC class I stress molecules MICA/B and HLA-E in intestinal epithelial cells (25, 35, 36), immune features that are both required for the development of villous atrophy. Additionally no tissue destruction was observed in HLA-DQ2 or HLA-DQ8 humanized mice that develop anti-gluten immunity (37–39). Only in recent years has it become clear that the interplay between gluten-specific CD4^+^ T cells and intraepithelial cytotoxic CD8^+^ T cells, as well as the simultaneous activation of innate immune pathways in distinct gut compartments, are required to cause villous atrophy observed in the active form of the disease (**Figure 1**).

## Key Roles of Transglutaminase 2 and HLA in the Initiation of the Gluten-Specific Adaptive Immune Response

The identification of HLA-DQ2 or HLA-DQ8 restricted CD4^+^ T cells in the lamina propria of CeD patients (26, 40) that preferentially recognize deamidated gluten peptides over native gluten peptides (41) stressed the connection between gluten, disease-associated HLA molecules and TG2 for the initiation of the pathogenic immune response. The generation of such a gluten-specific T cell response arises from the high affinity binding of gluten peptides post-translationally modified by TG2 to HLA-DQ2 or HLA-DQ8.

Two properties of gluten explain its ability to elicit a mucosal immune response. First, the high content of proline in gluten proteins makes the proteins resistant to degradation by intestinal proteases in the gut lumen (42). Second, the long undigested gluten-derived proteins are good substrates for the enzyme TG2, an ubiquitous and multifunctional enzyme expressed in many organs including the gut (43). It has been acknowledged for a long time that TG2 plays a key role in CeD pathogenesis as the enzyme is the target of autoantibodies that are highly-disease specific and used for the diagnostic work-up (2, 4). In addition, TG2 catalyzes the conversion of glutamine residues present in gluten peptides into glutamate (28–30). This deamidation process is key to initiate a pathogenic response in CeD as it promotes the generation of immunogenic peptides with negatively charged carboxylate residues that anchor with high affinity in the positively charged pockets of HLA-DQ2 or HLA-DQ8 binding grooves (5, 44).

In support of the role of TG2 in orchestrating mucosal immune responses to dietary gluten, TG2 is mostly found catalytically inactive in the intestine under physiological conditions but its expression and activity are increased in inflamed tissues and in cells with inflammatory stress (45). Interestingly, the administration of poly(I:C) (Polyinosinic:polycytidylic acid), which results in the rapid induction of villous atrophy that is the typical intestinal tissue injury observed in CeD patients, promotes TG2 activation (46). TG2 can be released into the gut lumen by small intestine enterocyte shedding, allowing TG2 to become catalytically active in the extracellular environment (47). This feature that allows the TG2 to be in close vicinity of gluten peptides could explain the formation of enzyme-substrate complexes between the two proteins that can bind to the B cell receptor of TG2-specific B cells, hence contributing to the generation of TG2-specific autoantibodies (24, 48, 49). In addition, using T84 enterocytic cell line as a model, it was shown that extracellular TG2 can be activated in a phosphatidylinositol-3-kinase-dependent mechanism by IFN-γ (50), the pro-inflammatory cytokine abundantly produced by gluten-specific T cells and released in the inflamed intestinal mucosa of CeD patients (31, 32). In accordance with a role for IFN-γ in TG2 activation, TG2 enzymatic activity can be triggered by the protein cofactor thioredoxin-1 (TRX) whose release from monocytic cells is also elicited by IFN-γ (51).

The requirement of gluten, predisposing HLA variant, TG2, and CD4 T cells to elicit the disease was formally demonstrated using a newly engineered DQ8-D^d^-villin-IL-15tg mouse model of CeD that develops villous atrophy upon gluten exposure (23). Using this model, we showed that villous atrophy, anti-deamidated gluten peptide antibodies and T_H_1 immunity recede on a gluten free diet and reoccur after gluten introduction. In addition, intestinal tissue destruction only occurred in mice carrying HLA-DQ8 and depletion of CD4^+^ T cells or administration of TG2 inhibitors in gluten-fed animals prevented the development of villous atrophy (23). Hence, the priming of a gluten-specific immune response depends on the coordinated interaction between gluten, the coeliac predisposing HLA-DQ8 molecule, activated TG2, and CD4^+^ T cells.

Interestingly, there is a gene dosage effect of the HLA-DQ2 or HLA-DQ8 allotypes in CeD whereby disease susceptibility depends on the HLA-DQ genotype and HLA homozygous individuals are at higher risk of developing the disease (52, 53). The haplotype HLA-DQ2.5 that binds and presents the full repertoire of gluten peptides, with many proline-rich α- and ω-gliadin-derived peptides that are protected from the degradation by gastrointestinal enzymes, confers a higher risk of developing CeD as compared to HLA-DQ8 that presents a smaller repertoire of immunogenic peptides more prone to proteolytic degradation and HLA-DQ2.2 that can only bind and present a few peptides (42, 54, 55). In addition, DQ2.5 has an increased ability to retain its peptide cargo as compared to DQ2.2. thanks to the presence of a polymorphism in the DQα chains allowing DQ2.5 to establish a hydrogen bond to the peptide main chain that stabilizes peptide-MHC complexes at the surface of antigen-presenting cells leading to sustained gluten peptides presentation to T cells (56). Interestingly, differences in the nature of HLA-DQ2.2 or HLA-DQ2.5-bound epitopes translates into a more diverse TCR repertoire generated in the context of HLA-DQ2.2, as compared to HLA-DQ2.5-mediated CeD and with a lower disease penetrance (57). Homozygosity for HLA-DQ2.5 that is linked to a heightened expression of HLA-DQ2.5 on the surface of antigen-presenting cells and increased antigen presentation is also more strongly associated with CeD as compared to heterozygosity (52, 55). Hence, the amounts of HLA-DQ-gluten peptides complexes correlates with the magnitude and breadth of the gluten-specific T cell response (52, 55). This suggests that CeD development and the ensuing intestinal tissue destruction will only occur when the T cell response has reached a certain magnitude to become pathogenic (35).

## CD4 T Cells and the Pro-Inflammatory Response

Under homeostatic conditions, ingested dietary antigens induce oral tolerance, a state of local and systemic immune ignorance against orally ingested innocuous antigens (58, 59). However, in patients harboring the CeD-predisposing HLA-DQ2 or HLA-DQ8 molecules, orally ingested gluten can initiate a gluten-specific pro-inflammatory T_H_1 response rather than a tolerogenic response (35). In both adults and children, these gluten-specific CD4 T cells produce high levels of the pro-inflammatory cytokines IFN-γ and IL-21, hallmarks of T_H_1 cells (32, 33, 60). As loss of oral tolerance to gluten is a preceding event for the development of villous atrophy in CeD patients (25, 32), much work has been done to uncover the mechanisms behind this loss of tolerance against gluten and how this pro-inflammatory T_H_1 CD4 T cell response leads to villous atrophy.

One of the early culprits implicated in promoting an inflammatory *vs* tolerogenic T cell response to gluten is the cytokine interferon-α (IFN-α). IFN-α is a Type-1 interferon that is produced by almost all cells as an innate response to viral infection (61). Among its many immune effector-promoting roles, it has been shown to drive pro-inflammatory dendritic cells activation as well as to promote the differentiation of CD4 T cells to the T_H_1 lineage (61, 62). The connection between IFN-α and CeD was made by Monteleone and colleagues, who identified a CeD-like enteropathy with villous atrophy and high intraepithelial lymphocytes infiltration in a chronic myeloid leukemia patient receiving an IFN-α treatment (63). The association between high expression of IFN-α and high levels of IFN-γ in CeD patients compared to controls suggested that IFN-α in CeD patients may be one factor leading to induction of a T_H_1 response against gluten. It remains unclear what directly is driving the increase in IFN-α production, but recent studies have also implicated viral infection as a driver for loss of oral tolerance.

While viral infections, such as with adenovirus or hepatitis C, have long been known to be associated with a higher risk of developing CeD (64), only recently have viral infections been mechanistically shown to induce loss of oral tolerance to gluten and dietary antigens. Using the Type-I Lang (T1L) reovirus strain, and murine norovirus (MNV) that both infect the gut, Bouziat and colleagues demonstrated that both viruses were capable of mediating T_H_1 responses to dietary antigens (18, 19). Type-1 IFN signaling was required for the blockade of peripheral regulatory T cells conversion while Interferon Regulatory Factor (IRF)1 expression was required for the induction of a T_H_1 immunity characterized by the differentiation of IL-12p40-producing dendritic cells, the production of gluten-specific IgG2c antibodies in the serum, TG2 activation in the proximal small intestine and a delayed type hypersensitivity reaction to gluten, all hallmarks of loss of oral tolerance to gluten in virus-infected HLA-DQ8 transgenic mice (18, 19). Taken together, these studies demonstrated that viral infections can be triggers for loss of oral tolerance towards dietary antigens and T_H_1-skewed responses to gluten.

Another major player implicated in the loss of oral tolerance to gluten is IL-15. The first signs that IL-15 may have been involved in the proinflammatory T_H_1 response to gluten came with the finding that IL-15 is heavily upregulated in the lamina propria of active CeD patients, the effector site where dendritic cells will encounter gluten peptides (65, 66). Using HLA-DQ8 transgenic mice that overexpressed IL-15 in the lamina propria and mesenteric lymph nodes (DQ8-D^d^-IL15tg mice) but not in the intestinal epithelium (38), we demonstrated that IL-15 overexpression in combination with retinoic acid altered the tolerogenic phenotype of intestinal dendritic cells and endowed them with a pro-inflammatory phenotype, hindering the development of Foxp3^+^ regulatory T cells and instead promoting the differentiation of IFNγ-producing T_H_1 cells. Additionally, these gluten-fed DQ8-D^d^-IL15tg mice displayed elevated levels of anti-gliadin and anti-TG2 antibodies, mimicking potential CeD patients who display a loss of oral tolerance and the development of a T_H_1 response to gluten in the absence of villous atrophy (38). In addition, IL-15 can block the immunosuppressive effects of TGF-β on CD4 and CD8 T cells by inhibiting Smad3-signalling and additionally render effector CD4 and CD8 T cells resistant to regulatory T cells-mediated suppression by activating PI3K-signaling (67, 68). Whether Foxp3^+^ regulatory T cells play an active role in dampening harmful immune responses to gluten in the small intestine remains poorly understood. Although it was shown that Foxp3^+^ regulatory T cells expand in the celiac lesion (69–71), it remains controversial whether regulatory T cells retain or loss their suppressive function (71–73). Moreover, regulatory CD4^+^ T cells specific for immunodominant gluten peptides haven’t been identified so far in the small intestine of genetically predisposed healthy individuals (74). Therefore, additional investigations are warranted to determine whether a regulatory response to gluten exists and whether an altered mucosal suppressive CD4^+^ T cell response to gluten contributes to CeD pathogenesis.

In the context of CeD, T_H_1 immunity is accompanied by the production of IFN-γ and IL-21 by mucosal gluten-specific CD4^+^ T cells (32, 33, 60). The idea of crosstalk between lamina propria and epithelium mediated by cytokines was put forward several years ago based on *in vitro* observations (75). Indeed, it had been shown that IFN-γ released by stimulated mucosal T cells was required for the optimal killing of human colonic epithelial cell lines in *ex-vivo* cytotoxic assays (76). In addition, the incubation of intestinal tissue specimens with the supernatants from gluten-stimulated T cell clones or with IFN-γ lead to epithelial cell damage, and the cytotoxic effect of the supernatants could be counteracted by the addition of neutralizing IFN-γ (77). We recently confirmed the requirement of IFN-γ for the activation of cytotoxic intraepithelial lymphocytes and the ensuing development of villous atrophy *in vivo* using a relevant mouse model of CeD (23). Although the exact mechanism underlying this effect remains to be uncovered, it has been shown that local production of IFN-γ can promote the upregulation of the non-classical MHC class Ib molecule HLA-E on epithelial cells (78, 79) hence potentializing the expression of the ligand for the activating NK receptor CD94/NKG2C present on cytotoxic intraepithelial lymphocytes during disease development. Although it has been shown that IL-21 can increase the cytotoxicity of human intraepithelial lymphocytes (80), the administration of an IL-21R blocking antibody in our mouse model didn’t reveal any significant direct role of IL-21 in promoting cytotoxic properties on intraepithelial lymphocytes but instead demonstrated that IL-21 and IFN-γ both play a role in the development of anti-deamidated gluten peptides antibodies (23). Interestingly, IL-15 can promote IL-21 production in lamina propria cells (81) reinforcing the idea of the involvement of a cytokine network in CeD.

While IFN-α, viral infections and IL-15 overexpression in the lamina propria can induce a loss of oral tolerance and a pro-inflammatory T_H_1 response to gluten, several studies in mice have demonstrated that the CD4 T cell response to gluten alone is nevertheless not sufficient to license intraepithelial lymphocytes and induce villous atrophy (23, 37, 38, 82). This is in accordance with observations in potential CeD patients who not only lack IL-15 and stress molecules expression on intestinal tissue cells, but also do not display an accumulation of intraepithelial cytotoxic T lymphocytes with an active killer phenotype, as seen in active CeD patients, despite the development of an inflammatory CD4 T cell response (25).

Interestingly, a distinct cytokine signature has been identified in the peripheral blood of treated CeD patients after oral gluten challenge (83, 84) or subcutaneous administration of T-cell stimulatory gluten peptides (85). Indeed, secretion of IL-2, IL-17A, TNF-α, IL-6 and IL-10 was detected as soon as 2h after gluten-re-exposure reflecting the rapid mobilization of gluten-specific memory CD4 T cells. Serum cytokine elevations, particularly IL-2 levels, correlated with the severity of acute digestive symptoms (83) and were specific to CeD (84, 86, 87), demonstrating a direct impact of gluten on the adaptive immune system in genetically susceptible individuals. Not only could serum cytokine release contribute to some extra-intestinal manifestations of CeD driven by inflammation, but it could be used as immune marker to diagnose and monitor the development of CeD.

## Innate Immune Response to Gluten and Epithelial Stress

The contribution of innate immunity to CeD pathogenesis was suggested by the observation that non-HLA genomic regions associated with CeD harbor genes involved in stress pathways and innate immunity (5). The activation of innate immune pathways in different gut compartments, in particular at the level of the lamina propria or in the intestinal epithelium, has a substantial impact on the adaptive immune responses taking place in those same compartments. These responses include the loss of oral tolerance to gluten and the associated induction of T_H_1 immunity, as well as the acquisition of lymphokine killer activity by intraepithelial lymphocytes, all of which contribute to disease development.

*In vitro* studies where gluten was used to stimulate duodenal biopsy samples, intestinal epithelial cells, monocytes, macrophages, and dendritic cells have shown that gluten may have innate immune stimulatory properties (88–94). Additionally, other molecules contained in wheat and related proteins could also drive immune cells activation, such as wheat amylase trypsin inhibitors (95, 96).

The gluten-derived α-gliadin peptide P31-43, which unlike the 33-mer (P55-87) and the 25-mer (P31-55) does not induce specific T cell responses in the celiac lesion, has been shown to activate innate immune pathways (90, 92, 97–99). However, the observed innate properties have been different across studies and we still need cross-validation. P31-43 peptide could induce enterocyte proliferation and actin rearrangements in an IL-15 and epithelial growth factor (EGF)-dependent manner, leading in particular to crypt hyperplasia, one of the characteristics of tissue remodeling seen in the celiac lesion (100–104). Other reported effects of gliadin on the innate immune system encompass cell structural changes, alterations in epithelial cells signaling, and induction of inflammatory and stress signals [reviewed in (100, 105)]. The finding that enterocytes from CeD patients have a stressed/inflamed phenotype and present a constitutive alteration in the intracellular vesicular trafficking provides an explanation as to why those cells are more sensitive to the effects of the P31-43 peptide (106–108). Indeed, due to sequence similarity between the P31-43 peptide and a region of hepatocyte growth factor regulated substrate (HRS) kinase - an essential protein involved in endocytic maturation-, P31-43 localizes in early endosomes and alters HRS-mediated maturation of early endosomes and the recycling pathway. The ensuing delayed vesicular trafficking leads to a reduction in the degradation of receptor tyrosine kinases including the receptor for EGF and promotes a sustained trans-presentation of IL-15 at the epithelial cell level [reviewed in (100, 105)].

Interestingly, a peptic-tryptic digest of gliadin or the P31-49-derived peptide can induce the upregulation of the expression of the stress-inducible MHC class I polypeptide-related molecules (MIC) *via* a pathway involving IL-15 (90). This is in accordance with the observation that intestinal epithelial cells in CeD patients express high levels of the MIC molecules (90, 109) and the non-classical MHC class I molecule HLA-E (79, 94). The expression of the inflammatory cytokine IL-15 can also be upregulated in whole biopsies, intestinal epithelial cells, or antigen-presenting cells from CeD patients upon gluten challenge or P31-49 derived peptide stimulation (65, 66, 78, 92, 110). The physiological consequences of the activation of immune pathways by gluten remain unclear given the fact that family members of CeD patients that lack an adaptive response to gluten, yet harbor the pre-disposing HLA-DQ2 and HLA-DQ8 and also show IL-15 upregulation in their intestinal compartment (25), retain normal intestinal morphology. However, the observation that, unlike active CeD patients, potential CeD patients -who display a gluten-specific adaptive immune response in the absence of tissue destruction- lack the innate epithelial stress response suggests that the alteration of the epithelial compartment is required for the development of villous atrophy (25). This observation is in accordance with the mechanism of epithelial cell destruction whereby activated intraepithelial TCRαβ lymphocytes mediate the killing of intestinal epithelial cells based on the recognition of stress signals such as non-classical MHC molecules and IL-15. As discussed in more detail below, the acquisition of innate-like properties by intraepithelial lymphocytes is also driven by IL-15 (111).

In addition to the epithelial upregulation of IL-15, most CeD patients display a chronic upregulation of IL-15 in the lamina propria (66). IL-15 plays a critical role in the lamina propria as it can impart dendritic cells to initiate the polarization of inflammatory T_H_1 responses and the loss of oral tolerance to gluten (38). This loss of oral tolerance to dietary gluten could also be triggered by type 1 interferons in individuals over-expressing IFN-α in lieu of IL-15 (63). Interestingly, the P31-43 peptide can trigger the expression of inflammatory mediators and increase cell death in a MyD88- and type 1 IFNs-dependent manner and this innate immune activation is enhanced by the TLR3 agonist poly(I:C) (112). This synergistic action of gluten peptides with poly(I:C) and the finding that poly(I:C) (46) or reovirus infection (19) promote the activation of TG2 suggests that multiple environmental hits can trigger and drive disease development. Because high levels of IL-15 can persist in a subset of patients on a gluten-free diet, it remains unclear what drives the excessive chronic upregulation of this innate cytokine. Similarly, both viral and bacterial infections could be the main source of type 1 interferons, yet how this expression is sustained remains to be determined (113).

The association between CeD susceptibility and single nucleotides polymorphisms in genes involved in microbial sensing has also pointed towards a role for bacterial microbes in triggering immune activation (5, 114). Many studies have noted differences in the microbiota composition between CeD patients, treated CeD patients on a gluten-free diet and healthy individuals [reviewed in (17)]. Gluten-degrading proteases produced by some opportunistic pathogens found in the duodenum of CeD patients such as *Pseudomonas aeruginosa*, can activate PAR-2 initiated inflammatory signaling pathways resulting in the expansion of intraepithelial lymphocytes (115). In addition, thanks to its elastase activity, *Pseudomonas aeruginosa* could contribute to the initiation of the disease by favoring the generation of immunogenic gluten peptides that can efficiently translocate through the intestinal barrier (116). *Neisseria flavescens*, abundantly present in the duodenal microbiome of CeD patients, could also contribute to the inflammatory response through its ability to endow a pro-inflammatory phenotype in dendritic cells (117). Dysbiosis is usually associated with a decrease in bacterial diversity and in the production of short-chain fatty acids such as butyrate, propionate and acetate that result from carbohydrate fermentation (118–121) and contribute to the maintenance of the gut homeostasis (122). Interestingly, a decrease in butyrate-producing bacteria such as *Bifidobacterium* or *Faecalibacterium prausnitzii* (123, 124) as well as alteration of the fecal metabolites patterns has been observed in children with CeD (125–127), yet these changes have not been observed in adult cohorts (128–130). These observations, together with the findings that genetically predisposed children carrying the HLA-DQ2 molecule present an altered gut microbiota composition, suggest that commensal bacteria could contribute early on to determining disease risk (131). In addition, because alterations in gut microbiota and fecal short-chain fatty acid composition can persist even when gluten is withdrawn, it is unclear whether dysbiosis could contribute to the initiation and enhancement of the disease or if changes in the microbiota reflect the ongoing local inflammation (15).

Overall, innate factors induced by gluten exposure and additional unknown triggers, perhaps of microbial origin, play a critical role in promoting the loss of oral tolerance to gluten and in altering intestinal epithelial cells that become the target of activated intraepithelial lymphocytes. However, studies comparing potential and active CeD patients as well as comparing mice expressing IL-15 in different gut compartments have shown that IL-15 and stress molecules overexpression in the epithelium need to be associated with adaptive immunity for villous atrophy to develop (23, 25).

## Destruction of Epithelial Cells by Cytotoxic Intraepithelial Lymphocytes

In addition to the T_H_1-skewed CD4 T cell response, another hallmark of CeD is the large accumulation of oligoclonal cytotoxic intraepithelial TCRαβ^+^ CD8^+^ lymphocytes (IE-CTLs) and TCRγδ^+^ intraepithelial lymphocytes (78, 109, 132). These cells, and not the gluten-specific CD4^+^ T cells, are the particular immune cell type thought to mediate the destruction of intestinal epithelial cells and directly lead to villous atrophy (23, 36). Their critical role in tissue destruction was not appreciated until the discovery that these cells are reprogrammed to express high levels of activating NK receptors and associated adaptor molecules. In healthy individuals, intraepithelial lymphocytes predominantly express the dimeric inhibitory CD94/NKG2A receptor with only low levels of the activating CD94/NKG2C and NKG2D receptors (78, 79, 133, 134). However, intraepithelial lymphocytes from CeD patients were found to undergo extensive NK cell-like reprograming, downregulating expression of the inhibitory CD94/NKG2A receptor and upregulating expression of the activating CD94/NKG2C and NKG2D receptors (78, 79, 133, 134). Furthermore, the CD94/NKG2C receptors in CeD patients are associated with the ITAM-bearing adaptor molecule DAP12, enabling cytokine secretion, proliferation and cytolytic activity in response to NK receptor ligands, even independently of TCR activation (79). The stress-inducible, non-classical MHC-like molecule HLA-E is the ligand for CD94/NKG2C and it is selectively upregulated on intestinal epithelial cells in CeD patients, allowing enterocytes to be targeted for killing by IE-CTLs (79). Upregulation of NKG2D on IE-CTLs in CeD patients, as well as upregulation of its adaptor molecule DAP10, was found to be directly caused by high levels of IL-15 on intestinal epithelial cells (109). This IL-15 mediated signaling not only upregulated NKG2D, but also acted in a co-stimulatory manner, synergizing with NKG2D signaling to enable TCR-independent cytolysis of targets expressing both IL-15 and the stress-induced NKG2D ligands MICA/B (109, 134, 135). MICA/B, which are highly expressed in the intestinal mucosa of CeD, enable enterocytes to be excellent targets for IE-CTLs-mediated destruction (90). Taken together, the reprograming of CD8^+^ intraepithelial lymphocytes into NK-like IE-CTLs with the ability to lyse target cells independently of TCR activation positions this cell type to be the direct mediator of tissue destruction in CeD.

It is important to note however that while no gluten-specific IE-CTLs have been identified in the intestinal epithelium of CeD patients, and IE-CTLs can kill intestinal epithelial cells in a TCR-independent manner, TCR specificity may still be playing a role in tissue destruction. Support for this idea comes from studies showing that inhibitory and activating NK receptor expression was associated with particular TCR specificities (133, 136). Additionally, signaling through CD94/NKG2C, NKG2D, and IL-15 receptors lowers the threshold for TCR activation (79, 109, 134). This co-stimulatory signaling could allow for low-affinity TCR-ligand interactions to activate TCR signaling, while under normal conditions the interaction would be too low affinity for activation. Evidence for this was shown in a study in which IL-15 expressing tumors were selectively controlled and killed by SIY-specific CTLs cells in a non-cognate but TCR-dependent fashion (137). Therefore, there still may be a potential role for TCR specificity among IE-CTLs as low affinity TCR interactions may be playing a part in tissue destruction along with TCR-independent cytolysis. Overall, although TCRαβ^+^ IE-CTLs are not gluten specific, they destroy specifically intestinal epithelial cells expressing IL-15 and ligands for activating NK receptors.

While the frequencies of TCRαβ^+^ CD8^+^ IE-CTLs decrease when gluten is excluded from the diet (35), the expansion of TCRγδ^+^ intraepithelial lymphocytes persists (132). Intriguingly, the composition of the tissue-resident TCRγδ^+^ compartment is irreversibly altered by inflammation with the depletion of innate-like Vγ4^+^/Vδ1^+^ intraepithelial lymphocytes and the expansion of gluten-sensitive IFN-γ-producing Vδ1^+^ intraepithelial lymphocytes (138). Although the exact role of the naturally occurring tissue resident TCRγδ^+^ intraepithelial lymphocytes that have both cytotoxic and tissue repair potential remains to be determined, their loss may lead to a defect in tissue healing and the protection against infections and tumors. Furthermore, the role of gluten-dependent production of IFN-γ by active CeD Vδ1^+^ intraepithelial lymphocytes remains elusive.

A subset of adults with CeD go on to develop refractory coeliac disease (RCD), a rare CeD complication in which patients have persistent severe villous atrophy despite being on a strict gluten-free diet (139, 140). One of the hallmarks of RCD is the expansion of aberrant intraepithelial cytotoxic lymphocytes that lack surface CD3 expression (sCD3^-^), express intracellular CD3 (iCD3^+^), and display a highly activated NK cell-like phenotype (140). These aberrant IE-CTLs develop from hematopoietic stem cell-derived CD103^+^sCD3^-^ IELs that encounter high levels of IL-15 and Notch signals in the gut epithelium and develop gain-of-function JAK1 or STAT3 mutations (141). Since both JAK1 and STAT3 are involved in IL-15 signaling, these gain of function mutations lead to heightened IL-15 signaling in aberrant iCD3^+^ IE-CTLs resulting in their expansion and survival through activation of anti-apoptotic signaling pathways (e.g. upregulation of anti-apoptotic factors Bcl-2 and Bcl-xl) (66, 142, 143). With IL-15 playing such a major role in the development of CeD and RCD, recent phase 2a clinical trials tested the impact of blocking IL-15 in CeD and RCD patients (144, 145). The trials did not show a significant difference in the primary clinical endpoints (improvement of the mucosal architecture in CeD and reduction in the proportion of aberrant intraepithelial lymphocytes in RCD). However, there were differences in some secondary endpoints, with treated RCD patients having fewer gastrointestinal symptoms and displaying less T cell receptor clonality than the placebo group (144), suggesting that blocking IL-15 may still be an option for treating RCD. There is a need to perform long-term follow-up studies in which the treatment duration will be significantly increased (duration of the anti-IL5 treatment in the published study was only ten weeks).

## Conclusion

**Figure 2:**
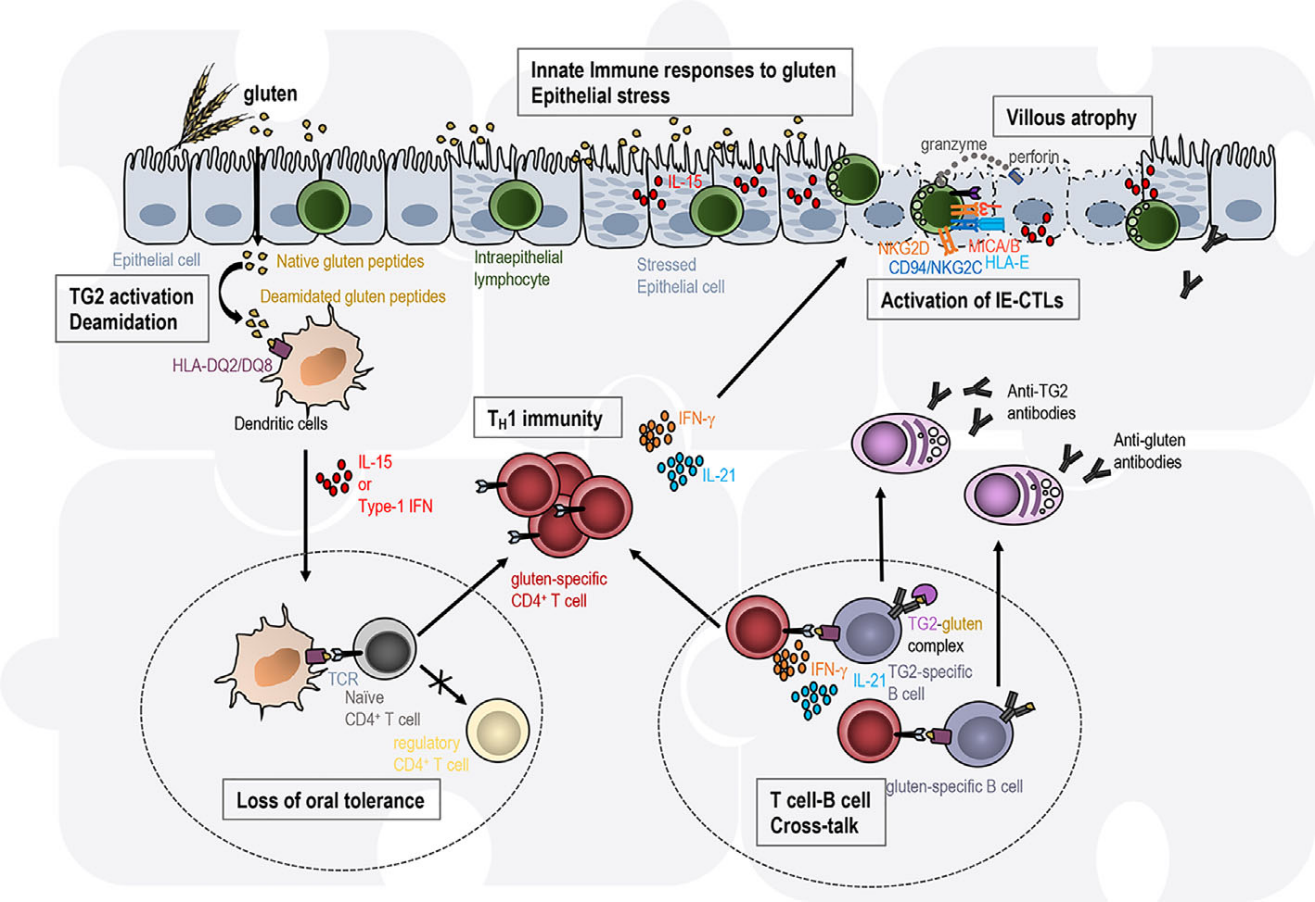
Intestinal tissue destruction in coeliac disease results from the interplay between several immune pathways in distinct gut locations. Transglutaminase 2 (TG2)-deamidated gluten peptides bind with high affinity to the disease-associated HLA-DQ2 or HLA-DQ8 molecules on antigen-presenting cells. In an inflammatory context (presence of IL-15, type 1 IFN), dendritic cells acquire a pro-inflammatory phenotype and migrate to the mesenteric lymph nodes (left circle with dashed line). They present gluten peptides to naïve CD4 T cells and promote T cell differentiation into T_H_1 effector T cells, while the induction of regulatory T cells involved in oral tolerance is abrogated. Anti-gluten CD4^+^ T cells directly secrete IFN-γ or IL-21. Anti-gluten CD4^+^ T cells are thought to provide help to gluten- and TG2-specific B cells in gut-associated secondary lymphoid organs (right circle with dashed line) leading to the production of IgA and IgG anti-gluten and anti-TG2 antibodies. In the presence of high IL-15 expression in the epithelium, intraepithelial lymphocytes acquire cytotoxic properties (activating NK receptors, release of the cytotoxic molecules granzyme B and perforin) and the ability to kill stressed epithelial cells expressing the ligands (HLA-E, MICA/B) for the NK receptors. Each of these immune events are required to promote coeliac disease but none of them individually is sufficient to promote intestinal tissue destruction. Hence the immunopathogenesis of coeliac disease is often presented as a jigsaw where each piece associated with one immune event needs to be connected to promote the disease.

As discussed throughout this review, both innate and adaptive immune responses possibly connected by a cytokine network contribute to the immunopathogenesis of CeD. Each of the described cell types and their known mechanism of action are required to promote CeD but none of them individually is sufficient to culminate in the full-blown disease characterized by intestinal tissue destruction and remodeling. Observations in mice and humans have suggested or demonstrated the requirement for the simultaneous activation of distinct pathways in different gut locations, some cell-cell interactions, and the existence of a crosstalk between the lamina propria and the epithelial compartments (**Figure 2**).

First, the simultaneous analysis of IE-CTLs and intestinal epithelial cells features in patients encompassing the spectrum of the disease - i.e. family members, potential CeD patients and active CeD patients- has led to the hypothesis that the combination of epithelial stress associated with high IL-15 expression in enterocytes and an anti-gluten adaptive immune response induced in the lamina propria in the presence of inflammatory mediators such as IL-15 is needed for the development of villous atrophy (25). The cooperation between epithelial IL-15 and CD4^+^ T cells to promote tissue destruction was also suggested by a study performed in mice overexpressing IL-15 in the gut epithelium and fed with the dietary antigen ovalbumin (146). The analysis of our DQ8-D^d^-villin-IL-15g mice modeling CeD patients upon gluten oral challenge unequivocally confirmed that the development of villous atrophy requires the concomitant presence of epithelial stress and anti-gluten adaptive immunity (23).

Next, in agreement with the findings that CD4^+^ T cells are required for the development of CeD, the prevention of villous atrophy in DQ8-D^d^-villin-IL-15tg mice treated with an anti-CD4 depleting antibody, anti-IFNγ depleting antibody or TG2 inhibitors concomitantly to gluten administration also underlined the existence of a cross-talk between T_H_1 immunity in the lamina propria and the activation of IE-CTLs (23).

In addition, a recent study using a mouse model of CeD lacking B cells has demonstrated that B lymphocytes are required for the development of villous atrophy and the induction of a killer phenotype in IE-CTLs (147). The mechanisms underlying the exact contribution of B-cell mediated immune responses to the immunopathogenesis of CeD remains to be determined. Most of our current understanding of the role of antibodies is based upon *in vitro* experiments. Although anti-TG2 antibodies can exert several effects, no consensus has been reached regarding their potential pathogenic role [reviewed in (148)]. While cytokines produced by B cells, that need yet to be identified, could contribute to the inflammatory process in the CeD lesion, B lymphocytes and/or plasma cells themselves could contribute to disease pathogenesis through their role as antigen-presenting cells. Indeed, plasma cells were found to be the most abundant gluten peptide MHC-expressing cells in the lamina propria of CD patients (149). Human studies have also suggested that gluten CD4^+^ T cells and B cells having internalized TG2-gluten complexes interact to promote the generation of anti-TG2 antibodies, whose formation rely on the presence of gluten (24, 48, 150, 151). This T cell-B cell crosstalk is supported by *in vitro* assays showing that transduced lymphoma B cells expressing HLA-DQ2.5 and binding catalytically active TG2 can activate gluten-specific hybridoma T cells in the presence of non-deamidated gluten peptides (151). However, whether this interaction also benefits CD4^+^ T cells by promoting their activation and expansion helping to reach the threshold needed to reach a pathogenic T cell response remains to be determined.

T cell clones have proven to be an invaluable research tool to gain insights into the immune mechanisms underlying CeD pathogenesis. Early analysis of CD4 T cell clones in celiac patients uncovered T_H_1-skewed gluten-specific CD4 T cells restricted to the disease-associated HLA-DQ2 and HLA-DQ8 molecules (26, 31, 40). Further interrogation of gut derived gluten-specific T cell clones helped to demonstrate that these clones respond to TG2-modified gluten peptides and that deamidation of these peptides leads to the strongest T cell response (29, 30). The usage of animal models of the disease combined with our ability to track gluten-specific T cells should help identify the intestinal location of the pathogenic cellular interactions described in this review, such as the B cell-T cell crosstalk (37, 152). Leveraging single-cell approaches technologies to study human samples could also help identify additional understudied cell subsets such as innate lymphoid cells [reviewed in (138, 153)] that could participate in the establishment and maintenance of the disease.

Altogether, CeD represents a perfect example of a multifactorial complex autoimmune disorder. The priming of a gluten-specific inflammatory immune response depends on the coordinated interaction between gluten, the celiac predisposing HLA-DQ molecule, activated TG2, and CD4^+^ T cells, while the alteration of epithelial cells expressing stress molecules, and the subsequent activation of IE-CTLs are all required to promote intestinal tissue destruction. How T lymphocytes in the lamina propria and cells present in the epithelial compartment communicate and where the cross-talk between distinct cell types occur is not yet understood and will certainly lead to the identification of signaling pathways that could potentially represent novel therapeutic targets.

## Author Contributions

All authors contributed to the article and approved the submitted version.

## Funding

This work was supported by a Young Investigator Award from the Celiac Disease Foundation to VA.

## Conflict of Interest

The authors declare that the research was conducted in the absence of any commercial or financial relationships that could be construed as a potential conflict of interest.
